# OCT biomarkers related to subthreshold micropulse laser treatment effect in central serous chorioretinopathy

**DOI:** 10.1186/s12886-022-02472-1

**Published:** 2022-06-07

**Authors:** Fang Zheng, Jingliang He, Zhitao Su, Ye Liu, Yufeng Xu, Lei Liu, Panpan Ye

**Affiliations:** 1 Eye Center, Second Affiliated Hospital, College of Medicine, Zhejiang University, No.88, Jiefang Road, Hangzhou, 310009 China; 2 Guangdong Eye Institute, Department of Ophthalmology, Guangdong Provincial People’s Hospital, Guangzhou, China

**Keywords:** Subthreshold micropulse laser, Central serous chorioretinopathy, OCT biomarkers, Choroidal thickness, RPE/inner choroidal alterations

## Abstract

**Background:**

To identify the OCT biomarkers related to the anatomical outcomes in eyes with central serous chorioretinopathy (CSCR) after subthreshold micropulse laser (SML) treatment.

**Methods:**

Patients with CSCR underwent SML were enrolled in this retrospective study. Only patients who underwent enhanced depth imaging optical coherence tomography (EDI-OCT) examination before and after SML were selected. Patients were divided into two groups based on whether subretinal fluid (SRF) absorbed or not after SML. Group 1 was the SRF resolved group, and Group 2 was the SRF non-resolved group. Factors including age and gender, duration of symptoms, CSCR history, the height of SRF at baseline, retinal pigment epithelium (RPE) /inner choroid alterations, as well as subfoveal choroidal thickness (SFCT) of the affected eye and the fellow eye before and after SML were recorded and compared between two groups. Longitudinal change of SFCT of a subgroup of patients were analyzed.

**Results:**

A total of 58 eyes of 58 patients were involved in this study. SRF of 31 eyes got completely absorbed, and SRF of 27 eyes was retained after SML. Logistic regression analysis revealed baseline SFCT of the affected eye (OR = 1.007, 95% CI: 1.001–1.012, *P* = 0.019) and RPE/inner choroid alterations (OR = 25.229, 95% CI: 2.890–220.281, *P* = 0.004) were correlated with SML efficacy. Thirty-three eyes of 33 patients were enrolled in the subgroup analysis. A significant difference of SFCT changes between two groups were demonstrated (*P* = 0.001). The difference of SFCT between baseline and three months after SML was also related to SRF resolution (OR = 0.952, 95% CI: 0.915–0.990, *P* = 0.014).

**Conclusion:**

Baseline SFCT, change of SFCT at 3-month after treatment, and RPE/inner choroid alterations were the OCT biomarkers related to SRF resolution after SML treatment.

## Background

Central serous chorioretinopathy (CSCR) is one of the leading causes of vision problems in the middle-aged population, characterized by subretinal fluid (SRF) with or without pigment epithelial detachment (PED) [1]. Although most acute CSCR is self-limited with a recurrence rate of 30 to 50%, about 15 ~ 20% of acute CSCR converted to chronic type [2–4], characterized by persistent SRF more than four months [1, 5] and retina pigment epithelium (RPE) atrophy, leading to permanent damage of photoreceptors. Treatment is recommended for chronic or recurrent CSCR, including photodynamic therapy (PDT), focal laser photocoagulation of leakage point on fluorescein fundus angiography (FFA) images, subthreshold micropulse laser (SML), oral diuretics, and systemic or local carbohydrase inhibitors [6]. Anti-vascular endothelial growth factor should be considered when secondary choroidal neovascularization (CNV) is developed [7, 8]. So far, no consensus exists regarding the management of CSCR. Among these treatment options, SML is the only one that does not cause functional damage or structural damage or side effects [9]. Due to the complications and lack of clear effect of focal laser, it has been used less and less in clinical practice [10]. Complex CSCR according to the new multimodal imaging based classification [11] often required PDT [12]. However, the shortage of Visudyne supply in China has limited the application of PDT for CSCR patients. Considering the self-limiting nature of acute CSCR, the current standard care for it is observation. However, as many studies indicated, even short period fluid accumulation in the subretinal space may cause a certain amount of damage to photoreceptors [13, 14]. In order to save photoreceptors from SRF damage, SML was chose to treat the acute CSCR by some clinicians based on its the safety nature. Compared with observation, acute CSCR treated with SML had better visual acuity and higher contrast sensitivity, and less risk of recurrence and progression into chronic version [15].

It is thought SML targets retinal pigment epithelium (RPE) and stimulates functional RPE to drain SRF into choroidal circulation [9]. One recent study showed the efficacy of SML is related to RPE function [16]: SML is effective for restoring macular anatomy and visual sensitivity in CSCR cases with mild or moderate RPE defects; in contrast, it has only a minimal outcome in CSCR eyes with severe RPE defects. Moreover, previous studies have shown that older age, higher choroidal thickness, and inner choroid attenuation are related to CSCR duration and visual recovery [17–19]. However, it is not clear whether these factors are related to anatomic outcomes after SML in CSCR.

The purpose of this study is investigating the optical coherence tomography (OCT) biomarkers related to SML efficacy in CSCR, with particular attention to choroid thickness, RPE alterations and inner choroid attenuation.

## Methods

Patients diagnosed with CSCR from November 2020 to January 2022 were enrolled in a retrospective study at Eye Center, Second Affiliated Hospital of Zhejiang University. The Institutional Review Board of the Second Affiliated Hospital of Zhejiang University approved the study. The study was performed in accordance with the tenets of the Declaration of Helsinki and compliant with the Health Insurance Portability and Accountability Act of 1996. The need for written informed consent was waived by the Second Affiliated Hospital of Zhejiang University because of the retrospective design and the use of de-identified patient data.

Patients of CSCR, either acute or chronic CSCR, who underwent SML treatment were selected. No patient had received direct CSCR treatments, including focal laser, PDT, and SML. All patients underwent FFA and OCT angiography (OCTA, OptovueRTVue XR 100; AVANTI, Inc) examination to rule out the presence of CNV. The FFA patterns of all patients involved in this study presented diffused leakage or leaking spots within 500 μm to the fovea. The SML treatment was performed by a single doctor (PPY) with a 577 nm micropulse laser mode (Quantel Medical). The laser parameter was set to a 5% duty cycle. The spot size was 160 μm. The exposure duration was 200 ms. A nine-spot matrix with non-spacing between two spots was chosen. On the micropulse mode, the threshold power was titrated at the arch area to show a light visible burn. And the power was doubled in the micropulse emission mode. The final laser power used for patients was around 400mW. SML was performed on the macular area with SRF. The total laser burns were between 150 and 200.

Macular raster OCT images (Spectralis OCT, Heidelberg Engineering, Inc., Heidelberg, Germany) were collected under enhanced depth imaging (EDI) mode before and after SML treatment. The exclusion criteria include previous retinal surgery history, ocular trauma, presence of CNV, coexistence of other retinal disorders such as pathologic myopia and diabetic retinopathy, and inability to obtain EDI-OCT images due to media opacity or significant eye movements.

Patients were categorized into two groups based on whether SRF was completely absorbed or not after SML. Group 1 is the SRF resolved group, and Group 2 is the SRF non-resolved group. The patients with EDI-OCT images taken at both one month and three months after SML treatment were enrolled in a subgroup analysis for investigating the longitudinal change of SFCT after SML.

**Fig. 1:**
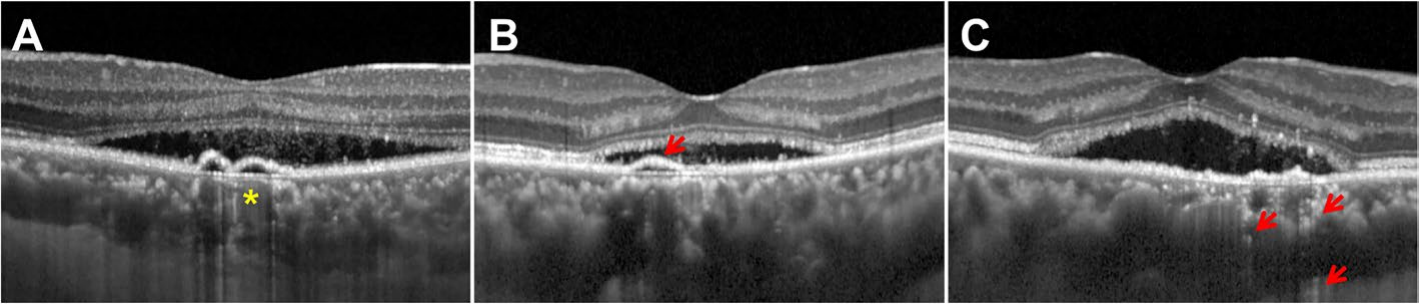
Demonstration of RPE/inner choroid alterations observed in the CSCR patients. **A** The OCT image showing inner choroid attenuation pushed by the underlying large choroid vessels (asterisk). **B** The OCT image showing irregular PED (red arrow). **C** The OCT image showing hyper-transmission defects (red arrows)

Subfoveal choroidal thickness (SFCT) and the height of SRF were measured by FZ using the build-in software of the instrument from the OCT scan through the fovea. If the choroidal-scleral boundary is ambiguous on the OCT images, the case is ruled out from the final analysis. The presence of RPE hyper-transmission defects, irregular PED, and/or inner choroid attenuation on OCT images were defined as RPE/inner choroid alterations (as shown in Fig. 1). The RPE/inner choroid alterations on baseline OCT images were evaluated by two independent graders (FZ and JLH). They were blinded to the grouping. After completing grading, the graders discussed disagreements and produced a consensus. The disagreement was adjudicated by a senior grader (PPY). Demographic information like age and gender, duration of symptoms, and CSCR history was recorded.

All data were described as mean ± SD. Chi-square test was used to analyze descriptive data. The continuous data was analyzed by an independent-sample T-test. The means of SFCT measured in different visits were compared with repeated measures analysis of variance (ANOVA). The factors related to the efficacy of SML were analyzed by the logistic regression model. The *P* value less than 0.05 was recognized as statistically significant. Statistical analysis was performed with IBM SPSS Statistics for Windows, Version 26.0 (IBM Corporation, Armonk, NY, USA).

## Results

A total of 58 eyes of 58 patients (47 male) with a mean age of 47.72 ± 8.53 years old were involved in this study. SRF of 31 eyes (53.45%) reached completely absorbed after SML (Group 1), and SRF of 27 eyes (46.55%) was retained after SML (Group 2). There was no difference in age, gender, and CSCR history between the two groups. SFCT of the affected eyes and in the fellow eyes was not significantly different between Group 1 and Group 2. And no significant difference was detected in baseline height of SRF between two groups. In Group 2, only one subject was graded as having no RPE/inner choroidal alterations. Statistic differences were found in the duration of symptoms and percentage of patients having RPE/inner choroid alterations between the two groups (Table 1). Logistic regression analysis revealed baseline SFCT of the affected eye (OR = 1.007, 95% CI: 1.001–1.012, *P* = 0.019) and RPE/inner choroid alterations (OR = 25.229, 95% CI: 2.890–220.281, *P* = 0.004) were correlated with the resolution of SRF. Other factors including age (OR = 1.051, 95% CI: 0.985–1.121, *P* = 0.131), gender (OR = 1.056, 95% CI: 0.283–3.945, *P* = 0.935), duration of symptoms (OR = 1.373, 95% CI: 0.965–1.952, *P* = 0.078), CSCR history (OR = 2.162, 95% CI: 0.779–6.659, *P* = 0.134), the height of SRF (OR = 1.002, 95% CI: 0.991–1.006, *P* = 0.435), and SFCT of the fellow eye (OR = 1.002, 95% CI: 0.997–1.007, *P* = 0.507) were not predictive factors of SML efficacy.

**Table 1:** Baseline characteristics of CSCR patients

	Group 1	Group 2	*P* value
Age (years)	46.13 ± 8.91	49.56 ± 7.82	0.538
Female/Total	6/31	5/27	0.953
Duration of symptoms (months)	2.42 ± 1.36	4.04 ± 3.85	0.026
Acute CSCR/Total	2/31	5/27	0.159
With CSCR history/Total	10/31	14/27	0.131
SRF height (μm)	187.84 ± 121.95	212.96 ± 124.42	0.942
SFCT of the diseased eyes (μm)	405.97 ± 112.24	511.81 ± 135.79	0.196
SFCT of the fellow eyes(μm)	372.82 ± 116.68	395.15 ± 115.21	0.888
RPE and inner choroid alterations/Total	15/31	26/27	0.000

**Fig. 2:**
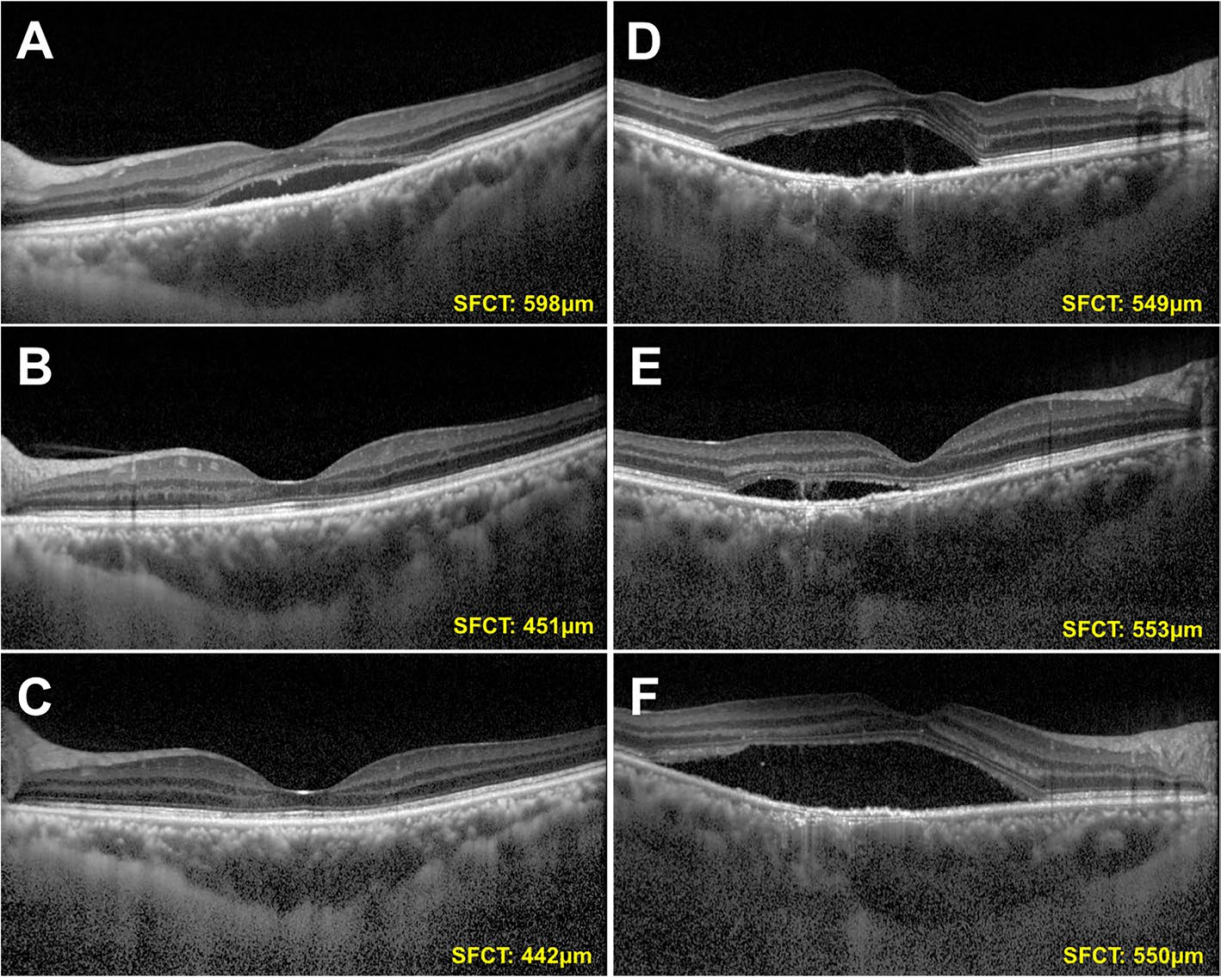
Demonstration of subfoveal choroidal thickness (SFCT) change after subthreshold micropulse laser (SML) in the different subgroups using enhanced depth imaging optical coherence tomography (EDI-OCT). **A** The EDI-OCT image of a patients before SML treatment in Group 1 showing no abnormality of RPE and a SFCT of 598 μm. **B** The EDI-OCT image of the patients at one month after treatment in Group 1 showing complete absorption of subretinal fluid (SRF) and a SFCT of 451 μm. **C** The EDI-OCT image of the patients at three months after treatment in Group 1 showing no recurrence of SRF and a SFCT of 442 μm. **D** The EDI-OCT image of a patients before SML treatment in Group 2 showing RPE hyper-transmission defects, irregular PED, inner choroid attenuation and a SFCT of 549 μm. **E** The EDI-OCT image of the patients at one month after treatment in Group 2 showing persistence of SRF and a SFCT of 553 μm. **F** The EDI-OCT image of the patients at three months after treatment in Group 2 showing increased in height of SRF and a SFCT of 550 μm

**Fig. 3:**
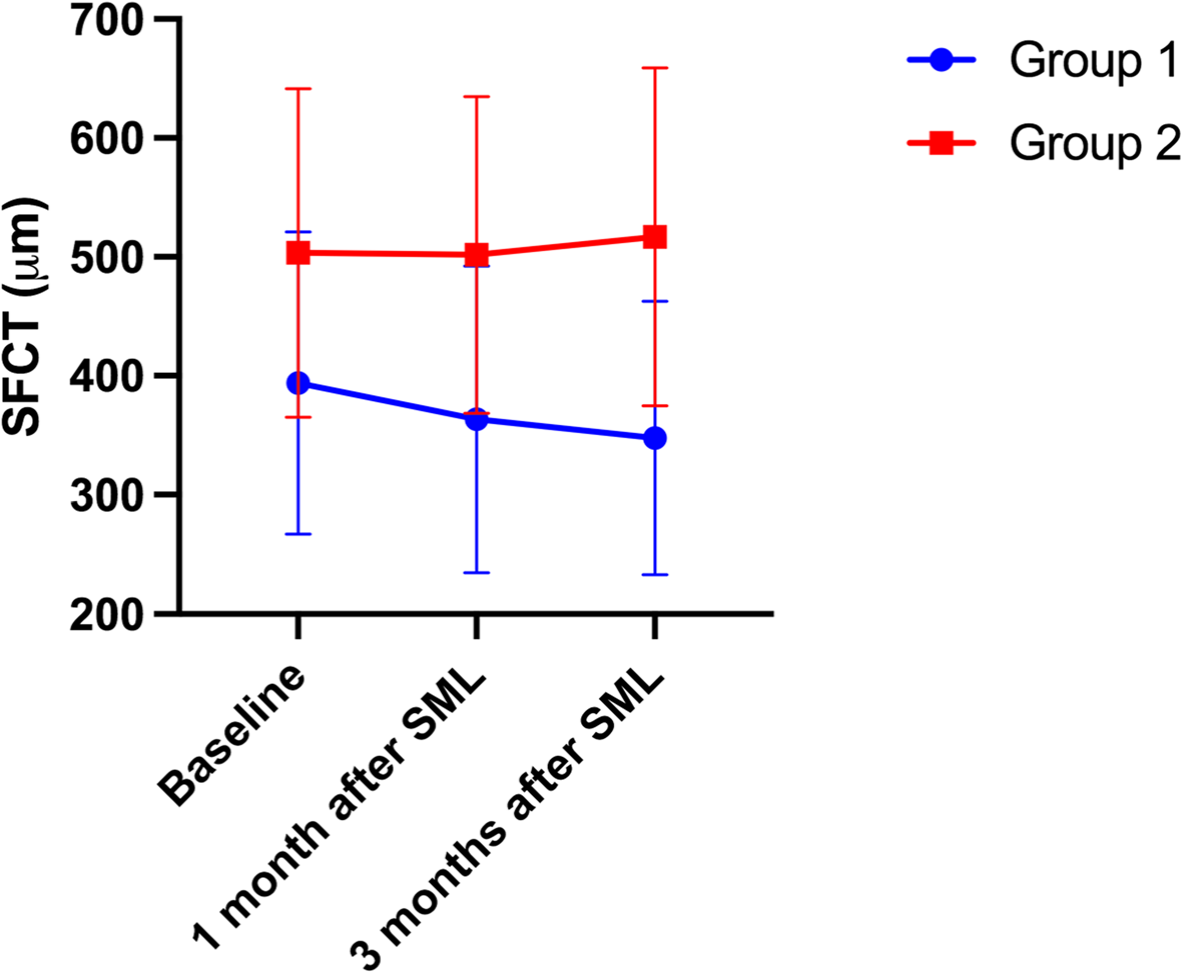
Plots showing subfoveal choroidal thickness (SFCT) change after subthreshold micropulse laser (SML) treatment in two groups

Thirty-three eyes of 33 patients with two post-SML EDI-OCT taken were enrolled in the subgroup analysis. In Group 1, the mean SFCT measurements of baseline, one month after SML and three months after SML were 394.06 ± 126.92 μm, 363.50 ± 128.58 μm, and 347.81 ± 114.70 μm, respectively; in Group 2, mean SFCT measurements remained stable with slightly increased at three months after SML (503.53 ± 138.14 μm, 501.71 ± 133.01 μm, 516.88 ± 142.25 μm) (Figs. 2, 3). Repeated measures ANOVA demonstrated a significant difference in serial SFCT changes between the two groups (*P* = 0.001). However, the changes of SFCT within groups were not statistically significant (All *P* > 0.05). The difference between baseline SFCT and 3-month SFCT was also correlated to anatomic outcomes after SML (OR = 0.952, 95% CI: 0.915–0.990, *P* = 0.014).

## Discussion

This study showed a 53% resolution rate of SRF after SML in CSCR patients. Baseline SFCT of the affected eye and the presence of RPE/inner choroid alterations were the OCT biomarkers related to SML efficacy. The decline of mean SFCT after SML was only noticed in Group 1. By comparison, mean SFCT of Group 2 got slightly increased. The change of SFCT after SML was also identified as a factor influencing the anatomic outcomes of SML in CSCR in the current study.

Unlike traditional laser photocoagulation, SML avoids scarring of RPE and retina by diffusing the heat to surrounding tissue. SML can stimulate SRF absorption by inducing reactions in RPE chromophores, including the possible production of heat shock protein [20]. SML has been shown to be effective in both acute and chronic CSCR. Superior visual rehabilitation was noted in SML-treated patients compared to observation in terms of BCVA, contrast sensitivity, and metamorphopsia in acute CSCR [15]. Arsan et al. demonstrated SML could significantly increase BCVA and decrease SRF in non-resolving CSCR patients [21]. Prasuhn et al. showed decreased central retinal thickness after SML in CSCR patients with persistent SRF either with or without secondary CNV [22]. However, compared with half-dose PDT, SML has a lower SRF complete resolution rate [23]. The PLACE trial, a multicenter prospective randomized clinical trial designed for chronic CSCR, showed 29% of patients with complete SRF resolution in the SML group and 67% of patients with complete SRF resolution in the half-dose PDT group at 7–8 months after the first treatment [23]. In our study, the resolution rate of SRF is 53% after a single SML, which is higher than PLACE trial. The reason might be both acute and chronic CSCR were involved in this study. And Group 1 had much shorter symptoms than group 2, indicating more acute CSCR got resolved after treatment. What’s more, the inappropriate laser setting and application of SML in PLACE trail were thought to be the reason for low resolution rate of SRF [24].

Both choroid and RPE contribute to the pathogenesis of CSCR [1]. CSCR belongs to the pachychoroid disease spectrum [25]. The choroid thickness of the affected eyes in CSCR is much thicker than the healthy eyes and the contralateral eyes [26]. Increased choroid thickness indicates hyper-permeability alterations of the choroid and can be recognized as the indicator of disease activity [1, 27]. Even though baseline SFCT was not significantly different between the two groups, it acted as a predictor of SRF resolution in our study. The thicker choroid of diseased eyes indicates a worse response to SML, which might be explained by the high activity of CSCR of these patients. Decreased SFCT was noticed after different treatments for CSCR [28–31]. Similar alterations of choroid thickness after SML treatment were also reported by other researchers [21, 22]. Compared with observation, choroidal thickness was significantly lower in the SML-treated group at eight weeks, sixteen weeks, and six months after treatment in acute CSCR [15]. In our study, decreased SFCT was only noticed in the SRF-resolved group (Group 1). The decrease of SFCT may indicate less activity of the disease and improvement of choroidal hyperpermeability after SML treatment. In Group 2, no decrease of SFCT was observed at one month or three months after SML treatment, indicating the prolonged high permeability of choroid, which might be the reason for the persistence of SRF in these eyes. Besides, we have found the change of SFCT at three months after SML was correlated with resolution of SRF. Another group also found that change of SFCT through twelve months was correlated with resolution of disease [32]. Our findings enhanced the importance of choroid in the pathology and prognosis of CSCR.

Diffuse RPE changes are often observed in chronic CSCR, including RPE detachment, RPE hypertrophy, and RPE atrophy [1]. The alterations of RPE in CSCR have receiving increasing attention in recent years. Color fundus photo, fundus autofluorescence, and OCT were commonly used to evaluate the function of RPE. A 2- disc diameter of RPE atrophy was defined as threshold to subclassify CSCR into simple or complex, according to a multimodal imaging-based CSCR classification system [11]. Diffuse RPE atrophy was described in cases of severe chronic CSC, possibly caused by long-term SRF persistent, phachychoroid vessels compression, and inner choroid ischemia [33]. If the area of diffuse atrophic RPE alterations more than five disc diameters at the macula, it can be defined as severe chronic CSCR [34]. Hypertransmission of RPE on OCT images indicates RPE attenuation or disruption [35]. By detecting pigment loss of the RPE in CSCR patients, a new imaging modality named dark-field SLO images can be used to predict the response to laser treatments [36]. As inner choroid has a direct influence on RPE, inner choroid attenuation may cause ischemia of RPE, which may lead to RPE dysfunction eventually. Since SML was designed to target RPE, RPE and inner choroid alterations might have a certain impact on the treatment effect. This is proven by the fact that only one out of twenty-seven eyes in Group 2 was graded as having no RPE and inner choroid alterations. The presence of RPE/inner choroid alterations was also shown to be a predictive factor related to insufficient response to SML treatment.

A previous study found that age and disease duration were risk factors for persistent SRF after SML in eyes with chronic CSCR [37]. But in our study, these two factors were not correlated to SML efficacy. Even symptom duration of Group 1 (2.42 ± 1.361) was less than that of Group 2 (4.04 ± 3.848) significantly, it is not a predictive factor for SRF resolution. The possible explanation is that both acute and chronic CSCR were involved in this study. And the rate of acute CSCR in group 1 was not significantly different from that in group2. Moreover, when taking other factors into account, the symptom duration became less important.

One limitation of this study is that many subjects only had one visit after receiving SML. So only 33 eyes got two EDI-OCT taken after SML and these eyes were selected in the subgroup analysis for investigating longitudinal SFCT change after the treatment. Another limitation of this study is using a single OCT image for choroidal thickness measurement. However, a study has demonstrated that no significant difference between SFCT and the mean of choroidal thickness in the central millimeter of cube scans by using swept-source OCT [38]. We have to clarify that the change of choroid we measured in the current study is limited to the central macular region. Whether the change of choroid thickness in a larger area influents treatment effect of SML still needs further investigation. In addition, functional measurements, including microperimetry and visual acuity of CSCR patients before and after SML, were not recorded in this study. The functional improvement after SML is worthy of being investigated in future studies.

## Conclusions

SML is effective for certain CSCR patients. Thicker baseline SFCT of the affected eye and the presence of RPE/ inner choroid alterations predict unsatisfied outcomes after SML. Failure to observe a decrease in SFCT after SML indicates the likelihood of SRF persistence.

## Data Availability

The datasets used and analyzed during the current study are available from the corresponding authors on reasonable request.

## Abbreviations

CSCR: Central serous chorioretinopathy
SML: Subthreshold micropulse laser
EDI: Enhanced depth imaging
OCT: Optical coherence tomography
SRF: Subretinal fluid
RPE: Retinal pigment epithelium
SFCT: Subfoveal choroidal thickness
PED: Pigment epithelial detachment
PDT: Photodynamic therapy
FFA: Fluorescein fundus angiography
CNV: Choroidal neovascularization
